# Low frequency cerebral arterial and venous flow oscillations in healthy neonates measured by NeoDoppler

**DOI:** 10.3389/fped.2022.929117

**Published:** 2022-11-28

**Authors:** Siv Steinsmo Ødegård, Hans Torp, Turid Follestad, Martin Leth-Olsen, Ragnhild Støen, Siri Ann Nyrnes

**Affiliations:** ^1^ Department of Circulation and Medical Imaging (ISB), The Norwegian University of Science and Technology (NTNU), Trondheim, Norway; ^2^ Children’s Clinic, St. Olavs Hospital, Trondheim University Hospital, Trondheim, Norway; ^3^ Department of Clinical and Molecular Medicine (IKOM), The Norwegian University of Science and Technology (NTNU), Trondheim, Norway

**Keywords:** cerebral hemodynamics, cerebroprotective, autoregulation, low frequency oscillations, cerebral short scale variability, Doppler ultrasound, NeoDoppler

## Abstract

**Background:**

A cerebroprotective effect of low frequency oscillations (LFO) in cerebral blood flow (CBF) has been suggested in adults, but its significance in neonates is not known. This observational study evaluates normal arterial and venous cerebral blood flow in healthy neonates using NeoDoppler, a novel Doppler ultrasound system which can measure cerebral hemodynamics continuously.

**Method:**

Ultrasound Doppler data was collected for 2 h on the first and second day of life in 36 healthy term born neonates. LFO (0.04–0.15 Hz) were extracted from the velocity curve by a bandpass filter. An angle independent LFO index was calculated as the coefficient of variation of the filtered curve. Separate analyses were done for arterial and venous signals, and results were related to postnatal age and behavioral state (asleep or awake).

**Results:**

The paper describes normal physiologic variations of arterial and venous cerebral hemodynamics. Mean (SD) arterial and venous LFO indices (%) were 6.52 (2.55) and 3.91 (2.54) on day one, and 5.60 (1.86) and 3.32 (2.03) on day two. After adjusting for possible confounding factors, the arterial LFO index was estimated to decrease by 0.92 percent points per postnatal day (*p* < 0.001). The venous LFO index did not change significantly with postnatal age (*p* = 0.539). Arterial and venous LFO were not notably influenced by behavioral state.

**Conclusion:**

The results indicate that arterial LFO decrease during the first 2 days of life in healthy neonates. This decrease most likely represents normal physiological changes related to the transitional period. A similar decrease for venous LFO was not found.

## Introduction

Preterm infants and other neonates in intensive care are vulnerable to brain injury caused by both hypo- and hyperperfusion (1), especially during the transitional period (2). Hemodynamic instability is usually considered detrimental, especially in patients with impaired cerebral autoregulation (2, 3), and has traditionally been associated with negative clinical outcomes across different time scales (4). However, emerging evidence implies that short scale variability in arterial pressure and cerebral blood flow (CBF) may protect against acute challenges to cerebral perfusion, including hypovolemia and hypotension (4).

Low frequency oscillations (LFO), occur in arterial and venous CBF at approximately 0.1 Hz (5–14). Studies indicate that LFO represent a neuroprotective mechanism (1, 15, 16), influenced by metabolic, neurogenic and myogenic control systems (17). In adults, LFO in cerebral oxygenation measured with near-infrared spectroscopy (NIRS) appear to be related to increased reactivity of the microvasculature and, potentially, increased compliance and autoregulatory capacity (18). LFO in arterial CBF have been observed in both preterm (5–14) and term (5, 7, 8, 11, 12) neonates, but the significance in these age groups is not known. In neonates, LFO in venous CBF have not yet been described in publications. In adults, LFO in venous CBF likely represent a venous autoregulatory mechanisms (19, 20).

Non-invasive, bedside monitoring systems that can measure CBF continuously may provide important information about cerebral hemodynamics in patients with increased risk of neurologic injury (1). NIRS in combination with invasive blood pressure (BP) measurement, has been used to identify individualized optimal mean arterial pressure (MAP) for cerebral perfusion in infants with hypoxic-ischemic encephalopathy during therapeutic hypothermia (21). Time spent outside optimal MAP was associated with more severe magnetic resonance (MR) abnormalities (21). In piglets, NIRS in combination with invasive BP demonstrated that LFO can be used to find the optimal BP for pressure autoregulation (22).

To recognize pathological flow patterns, knowledge on physiological fluctuations of CBF needs to be established. NeoDoppler is a new, non-invasive plane wave ultrasound (US) system that allows for continuous monitoring of arterial and venous CBF velocities at several depths simultaneously. In this study we aimed to measure cerebral hemodynamics with NeoDoppler on the two first days of life in healthy term born neonates. A secondary aim was to assess whether LFO were present in both arteries and veins, and if they were influenced by postnatal age (PNA) and behavioral state (asleep or awake).

## Materials and methods

A convenience sample of 37 randomly selected healthy infants born after an uncomplicated pregnancy between gestational ages (GA) 37° and 42° weeks at the maternity ward at St. Olavs Hospital, Trondheim, Norway between January 15, and August 28, 2019 was included. Written informed consent was obtained from the parents of the neonates. Neonates with any medical problem, including need for phototherapy or interventions for low blood glucose, were excluded. A doctor and a nurse in the research team carried out the inclusion and data collection, and recordings were done during the first 2 days of life. Clinical parameters were obtained from medical records. Peripheral oxygen saturation (left or right foot) and heart rate (HR) were measured at inclusion with the Intellivue MP40 Patient Monitor (Phillips). The behavioral state of the infants during NeoDoppler recordings was observed and categorized as asleep (quiet- and active sleep) and awake (quiet awake and feeding). The Regional Committee for Medical and Health Research Ethics, REC Central (Reference 2017/314), The Norwegian Directorate of Health (Reference: 17/15181-11) and The Norwegian Medicines Agency (Reference 19/05458) approved the study.

The non-invasive NeoDoppler system is based on plane wave transmission and was developed by the US group at The Norwegian University of Science and Technology (NTNU). The probe housing was developed in cooperation with a product design company (Inventas, Trondheim, Norway). The user interface, probe housing (Tubifast 2-way stretch), probe fixation, positioning, validation and safety data have been described previously in a proof-of-concept paper (23). In this, NeoDoppler was found feasible and safe to use on 14 preterm neonates and 11 sick term infants (23).

**Figure 1:**
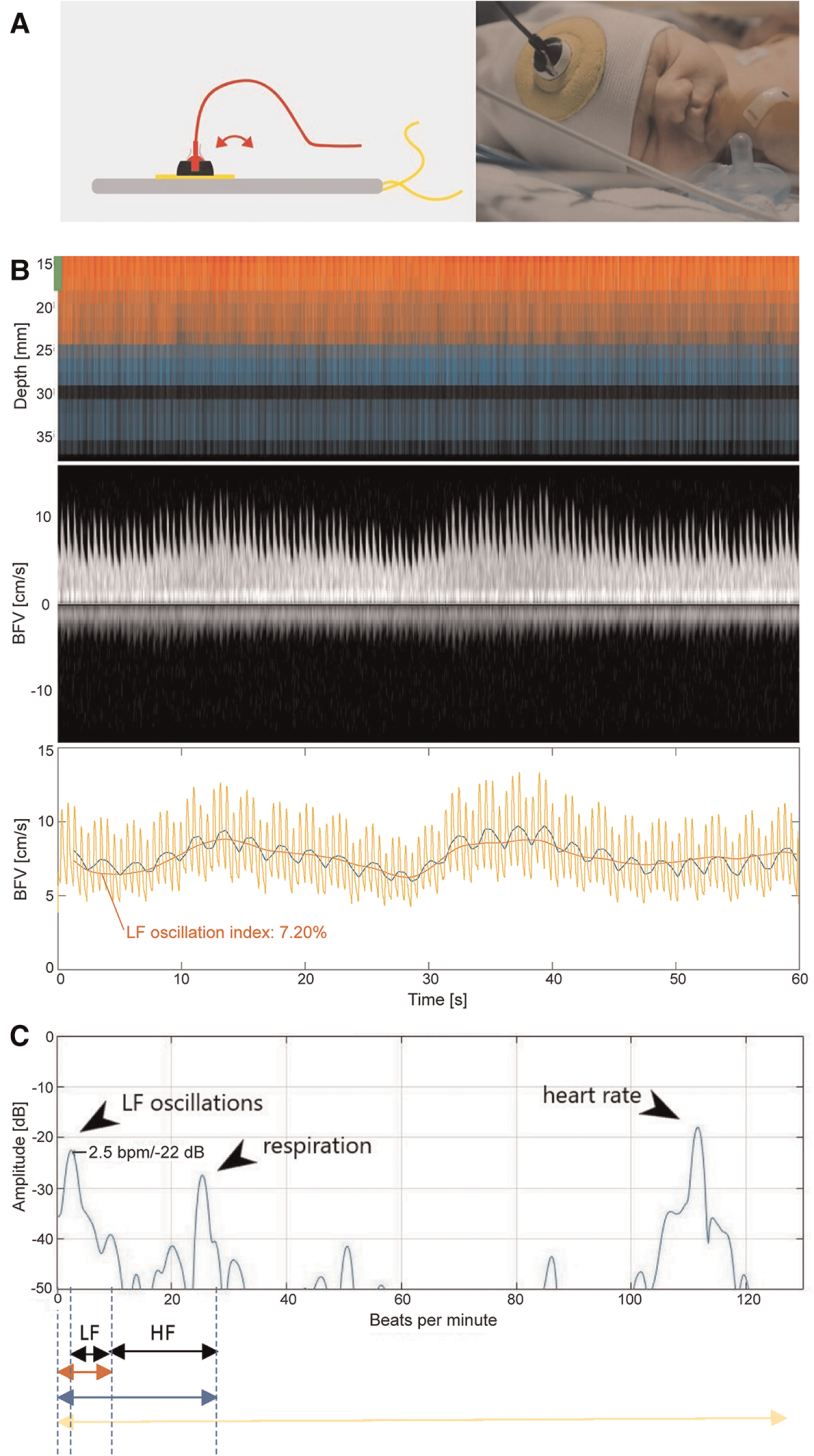
Low frequency oscillations recorded with NeoDoppler. (**A**) The soft elastic hat placed on the neonate's head is shown in the picture on the right. The probe holder (yellow more robust fabric with a white plastic cup) and ultrasound probe (in black) is placed over the anterior fontanelle. The left picture shows how the ultrasound probe can be adjusted in the probe holder (shifted slightly horizontally, vertically or in a different angle) to optimize the Doppler signal. (**B**) Upper panel: Demonstrates the NeoDoppler M-mode of cerebral blood flow in different depths, with time of recording along the *x*-axis and depth of recording along the *y*-axis (60 s on all images). (**B**) Middle panel: Shows the Doppler spectrum for the chosen sample volume (5 mm). The low frequency oscillations (LFO) are the slow changes in blood flow velocity (2.5 cycles/min) that can be seen visually. (**B**) Lower panel: The Doppler velocity curve (yellow) derived from the Doppler spectrum in the (**B**) upper panel, with the same time scale. The *y*-axis represents velocity (cm/second). The blue curve is the sum of the Doppler velocity mean value, low frequency- and high frequency oscillations. The red curve is the sum of the mean value and LFO. The LFO index (here 7.2 percent), is the standard deviation in percent of the mean blood velocity value over the full one-minute recording. (**C**) The frequency spectrum of the Doppler velocity curve, is another way to illustrate the variations in the cerebral blood flow. The beats/min are seen along the *x*-axis and amplitude (decibel) along the *y*-axis. The first peak at 2.5 beats/min, corresponding to 0.04 Hz (2.5/60 s), represents LFO. This slow periodic cycling is superimposed on the rhythm of other physiological parameters such as respiration (the second peak around 25 beats/min) and heart rate (peak around 115 beats/min). These three main periodic waves in the frequency spectrum correspond to the three curves in (**C**), as demonstrated with the red (LFO) blue (respiration) and yellow (heart rate) arrows below the frequency spectrum.

In short, the US probe is a single element 8-MHz transducer. It is a multigated Doppler, which measures bloodstream velocity in several depths simultaneously in a cylindric area down to approximately 35 mm (width 10 mm). During recording the NeoDoppler spectrum is displayed on a bedside screen. The sample volume indicates the depth where the pulsed-wave Doppler curves is obtained. If the wide sound beam picks up several arteries within the selected sample volume, the maximum velocity trace will represent the vessel with the highest velocity. Signals from veins and arteries can be distinguished from each other by the characteristics of the Doppler spectrum. No angle correction is performed. Trend curves, including HR, peak systolic velocity (PSV), mean velocity (Vmean) resistive index (RI) and pulsatility index (PI), were visualized on a separate bedside screen. Simple commands such as start/stop, and registration of events can be done during recording. Selection of depth, sample volume gain, velocity scale, and wall filter can be performed during the recording or by post-processing. An in-house software developed in MATLAB (MathWorks® R2017a) was used for data processing. The velocity measurements were averaged over one-minute. Processing time for one-minute recording was 1.08 s. This included filtering, spectrum analysis, automatic tracing of velocity curve, and calculation of all tabulated values. Visual evaluation of fluctuations in arterial and venous CBF was also performed. High grade venous fluctuations were defined in accordance with Ikeda et al. (24). An example of the Color M-mode display and the corresponding Doppler output are shown in Figure 1B.

The NeoDoppler probe, the fixation and positioning of the probe are shown in Figure 1A. The current prototype of the NeoDoppler US probe shown in Figure 1A, is fixated to the neonate's head supported by a cap with sticky silicon. The skin was examined after removal of the probe and fixation accessory to assess whether any local adverse effects had occurred. The neonates were either monitored in bed, during breastfeeding and/or in one of the parent's laps during examination. The parents were given a questionnaire regarding the feasibility of the probe and the fixation system.

### General automated analysis of the arterial Doppler signal: The full dataset

Successive recordings and pauses of 1 min were performed for 2 h during each of the 2 days. A tracing algorithm was used to trace the Doppler curve. For each recording, the arterial signal with the highest velocity and signal to noise ratio within the depth range from 1.8 to 38 mm was automatically selected for analysis (sample volume 4.8 mm). The quality of the US recordings was determined by a correlation coefficient of the velocity waveform between two consecutive heart beats, averaged over 1-min (>0.8 categorized as good). For arterial signals, all recordings were analyzed in a batch process without any user interactions.

Previous studies have used different definitions of LFO in neonates, ranging from 0.02 to 0.2 Hz (5–14). In this paper, LFO were defined as velocity oscillations in the frequency range 0.04–0.15 Hz and high frequency oscillations were defined as 0.15–4 Hz. A case demonstrating LFO is illustrated and explained in Figures 1B,C. The LFO is extracted from the velocity curve by a bandpass filter (2nd order Butterworth filter). The measured Vmean, as well as the LFO magnitude depend on the size of the vessel and the Doppler insonation angle. To avoid these dependencies, an LFO index was calculated as the coefficient of variation of the filtered curve, i.e., the standard deviation in percent of the mean blood flow velocity value over 1 min. A similar, but manual method for LFO measurement was presented in a study by Ferrari et al. (7).

### Manually sorted samples: To study the effect of behavioral state, imaging depth and vein signal characteristics

To analyze the effect of behavioral state, six recordings obtained during sleep and four recordings obtained during wakefulness from each infant were manually selected for analysis based on registration of state during monitoring. The study participants were mostly asleep, particularly on study day one. To investigate the effect of depth in the same selected samples, arterial signals were obtained from the depth of best quality most proximal and distal to the US probe respectively.

In these selected one-minute recordings, venous signals were also assessed. Due to limited available signals, venous data was obtained from one depth in each recording. When assessing venous signals an autogain function, where the gain was determined by the average background noise level in the Doppler spectrogram, was used to optimize the tracing prior to statistical analyses. If the tracing did not appear optimal after visual inspection, the velocity scale, wall filter and gain were manually adjusted.

This latter approach was also used for assessing the reproducibility of arterial velocity measurements, which was assessed from a random sample of five recordings in each of the four groups (asleep day one, asleep day two, awake day one and awake day two) with two depths from each recording, yielding 40 measurements in total. The data were reanalyzed by the same investigator (SSØ) after 17 weeks, and by a second investigator (ML-O), giving complete sets for intra- and interobserver variability.

### Statistical analyses

The statistical analyses were performed using IBM SPSS Statistics version 25 (IBM, New York, USA). Linear mixed models (LMM) were used to analyze the association between each of the velocity measurements: LFO index, PSV, Vmean, RI, PI and the explanatory variables (included as fixed effects) GA, PNA, behavioral state (asleep or awake), imaging depth and sex. In addition, univariable LMM analyses were performed, where day and state respectively were included as a fixed effect. Separate analyses were done for arterial and venous signals. The association between the velocity measurements and imaging depth did not seem to be linear; these measurements were therefore categorized into four categories for arterial signals and three for venous signals. A fixed effect for the interaction between PNA and behavioral state was included but turned out to be not statistically significant for any of the velocity measurements. Therefore, only results from LMM with main effects are presented. The correlation between repeated recordings provided by the same individual was accounted for by including subject-specific random intercepts. For the arterial signals, recording-specific intercepts nested within subjects were also included, since data from two depths were extracted from each recording. The normality assumption of the residuals was assessed by visual inspection of quantile-quantile (QQ) plots. The velocity measures from venous signals were log-transformed to make the distribution of the residuals closer to the normal distribution. To give some protection against false positive findings due to multiple testing, *p*-values <0.01 were considered statistically significant.

The intraclass correlation coefficient, based on a two-way random effects model and the absolute agreement type, was estimated to evaluate intra- and interobserver variability.

## Results

Out of 37 study participants, one was excluded due to respiratory illness. Data from two participants on study day one (behavioral state had not been assessed) and three participants on study day two (jaundice with need for phototherapy) were excluded from the statistical analyses. The 36 included neonates (58.2% boys) had a mean GA of 40 weeks (range 38–42), mean birth weight of 3,622 g (range 2,850–4,630). Mean APGAR scores at 1, 5 and 10 min were 9 (range 5–10), 10 (range 8–10) and 10 (range 9–10), respectively. The proportion of cesarian and instrumental deliveries were 27.8% and 13.9%, respectively. Mean age was 24 h (range 4–39) on study day one and 48 h (range 28–61) on study day two. All infants had a normal oxygen saturation (mean 98%, range 95–100), heart rate (mean 121 beats/minute, range 94–148), systolic BP (mean 74 mmHg, range 43–110), diastolic BP (41 mmHg, range 22–82) and MAP (mean 53 mmHg, range 34–93) at start of recording on study day one. The mean increase was 2.5 mmHg (SD 15.4,) for systolic BP, 4.3 mmHg for diastolic BP (SD 16.5) and 3.6 mmHg (SD 15.1) for MAP.

### Results from the general automated analysis: Including feasibility

The arterial velocity measurements are summarized in Table 1. The total number of recordings included was 1,222 on study day one and 1,148 on study day two, with a total recording time for all infants of 39.5 h. Number of hours with data quality >80% were 25.4 (64.3%; Table 1). PSV, Vmean, RI and PI are calculated per heartbeat. The LFO index was calculated per 1 min recording. The number of samples in Table 1 indicates the number of calculations for each velocity measurements, after exclusion of heart beats and recordings of too low quality (less than 80%).

**Table 1:** Cerebral hemodynamics in healthy neonates.

	All US recordings Day 1 (*n* = 1,222)			All US recordings Day 2 (*n* = 1,148)		
	Mean (SD)	Median (IQR)	*N* samples	Mean (SD)	Median (IQR)	*N* samples
LFO index (%)	7.03 (2.98)	6.52 (4.86–8.63)	815	5.98 (2.06)	5.69 (4.53–7.12)	701
PSV (cm/s)	8.82 (2.98)	8.31 (6.79–10.50)	87,410	9.97 (3.09)	9.60 (7.77–12.17)	71,672
Vmean (cm/s)	5.70 (2.07)	5.29 (4.27–6.79)	87,410	6.27 (1.98)	6.04 (4.82–7.62)	71,672
RI	0.63 (0.07)	0.63 (0.58–0.67)	87,410	0.64 (0.07)	0.63 (0.59–0.68)	71,672
PI	0.99 (0.21)	0.97 (0.85–1.10)	87,410	1.03 (0.21)	1.00 (0.89–1.13)	71,672

IQR, interquartile range; LFO, low frequency oscillations; *N* samples, number of calculations for each velocity measurements; PI, pulsatility index; PSV, peak systolic velocity; RI, resistive index; SD, standard deviation; US, ultrasound; Vmean, mean velocity.

### Results from the manually sorted recordings

The arterial and venous velocity measurements sorted by examination day and behavioral state are presented in Table 2. The multivariable analyses are presented in Tables 3–5, demonstrating the association between cerebral hemodynamics and velocity measurements, imaging depth and birth mode.

**Table 2:** Cerebral hemodynamics by postnatal age and behavioural state.

Day	Day one	Day two	Day one—day two
**Arterial [mean (SD)]**	***n* = 320**	***n* = 340**	**Mean (95% CI), *p*-value**
LFO index (%)	6.52 (2.55)	5.60 (1.86)	1.03 (0.63 to 1.42), <0.001
PSV (cm/s)	8.97 (2.92)	10.23 (4.06)	−1.03 (−1.50 to −0.57), <0.001
Vmean (cm/s)	5.71 (1.95)	6.44 (2.42)	−0.63 (−0.91 to −0.34), <0.001
RI	0.63 (0.07)	0.62 (0.06)	0.01 (0.00 to 0.02), 0.040
PI	1.01 (0.19)	1.00 (0.18)	0.02 (0.00 to 0.04), 0.076
**Venous median (IQR)**	***n* = 148**	***n* = 156**	***p*-value**
LFO index (%)	3.02 (2.11–4.96)	2.84 (2.03–4.02)	0.075
PSV (cm/s)	7.04 (4.17–12.63)	4.95 (2.82–8.53)	<0.001
Vmean (cm/s)	6.55 (2.83–8.53)	4.51 (3.53–11.56)	<0.001
RI	0.19 (0.14–0.27)	0.17 (0.13–0.22)	0.003
PI	0.21 (0.15–0.30)	0.19 (0.14–0.25)	0.001
**State**	**Asleep**	**Awake**	**Asleep—awake**
**Arterial [mean (SD)]**	***n* = 571**	***n* = 89**	**Mean (95% CI), *p*-value**
LFO index (%)	6.07 (2.29)	5.88 (2.13)	0.28 (−0.32 to 0.88), 0.360
PSV (cm/s)	9.56 (3.63)	10.03 (3.45)	−0.38 (−1.06 to 0.30), 0.275
Vmean (cm/s)	6.02 (2.24)	6.54 (2.13)	−0.42 (−0.83 to 0.00), 0.050
RI	0.63 (0.06)	0.60 (0.07)	0.02 (0.01 to 0.03), <0.001
PI	1.02 (0.18)	0.93 (0.20)	0.06 (0.03 to 0.09), <0.001
**Venous [median (IQR)]**	***n* = 276**	***n* = 28**	***p*-value**
LFO index (%)	2.89 (2.08–4.28)	3.64 (2.31–5.80)	0.092
PSV (cm/s)	5.60 (3.14–20.28)	6.48 (4.73–12.44)	0.695
Vmean (cm/s)	5.01 (2.83–9.47)	5.94 (2.83–9.47)	0.767
RI	0.17 (0.13–0.23)	0.19 (0.16–0.22)	0.048
PI	0.19 (0.14–0.27)	0.22 (0.15–0.26)	0.061

Linear mixed models were used to evaluate the association between velocity measurements and respectively day and state. Venous data are log-transformed. Mean difference and 95% confidence intervals are not included for log-transformed data. CBF, cerebral blood flow; IQR, interquartile range; LFO, low frequency oscillations; N samples, number of calculations for each velocity measurements; PI, pulsatility index; PSV, peak systolic velocity; RI, resistive index; SD, standard deviation; US, ultrasound; Vmean, mean velocity.

**Table 3:** Results from a linear mixed model to evaluate the association between cerebral hemodynamics and gestational age, postnatal age, sex, and behavioral state.

Measurements	Gestational age (weeks)	Postnatal age (days)	Sex (boy = ref)	State (awake = ref)
Arterial	Estimate (95% CI)	Estimate (95% CI)	Estimate (95% CI)	Estimate (95% CI)
LFO index (%)	0.14 (−0.18 to 0.45) *p* = 0.396	−0.92 (−1.31 to −0.53) *p* < 0.001	−0.82 (−1.57 to −0.06) *p* = 0.035	0.15 (−0.43 to 0.73) *p* = 0.606
PSV (cm/s)	−0.27 (−0.92 to 0.38) *p* = 0.409	1.07 (0.62 to 1.51) *p* < 0.001	0.32 (−1.32 to 1.95) *p* = 0.698	−0.29 (−0.91 to 0.33) *p* = 0.361
Vmean (cm/s)	0.02 (−0.38 to 0.42) *p* = 0.920	0.64 (0.36 to 0.91) *p* < 0.001	0.54 (−0.48 to 1.56) *p* = 0.292	−0.36 (−0.74 to 0.03) *p* = 0.068
RI	−0.03 (−0.04 to −0.01) *p* < 0.001	−0.01 (−0.01 to 0.00) *p* = 0.128	−0.03 (−0.06 to 0.00) *p* = 0.027	0.02 (0.01 to 0.03) *p* < 0.001
PI	−0.07 (−0.11 to −0.04) *p* < 0.001	−0.01 (−0.03 to 0.01) *p* = 0.227	−0.11 (−0.19 to −0.02) *p* = 0.016	0.06 (0.03 to 0.08) *p* < 0.001
**Venous (log-transformed)**				
LFO index (%)	0.09 (0.01 to 0.17) *p* = 0.038	−0.04 (−0.17 to 0.09) *p* = 0.539	−0.04 (−0.23 to 0.14) *p* = 0.638	−0.18 (−0.39 to 0.04) *p* = 0.103
PSV (cm/s)	0.00 (−0.14 to 0.14) *p* = 0.989	−0.27 (−0.40 to −0.14) *p* < 0.001	0.09 (−0.27 to 0.45) *p* = 0.620	−0.14 (−0.33 to 0.06) *p* = 0.173
Vmean (cm/s)	0.00 (−0.14 to 0.15) *p* = 0.953	−0.25 (−0.38 to −0.11) *p* < 0.001	0.08 (−0.29 to 0.45) *p* = 0.656	−0.12 (−0.32 to 0.09) *p* = 0.253
RI	0.00 (−0.07 to 0.07) *p* = 0.931	−0.13 (−0.21 to −0.05) *p* = 0.002	0.01 (−0.16 to 0.17) *p* = 0.936	−0.19 (−0.32 to −0.06) *p* = 0.004
PI	−0.01 (−0.08 to 0.07) *p* = 0.905	−0.16 (−0.25 to −0.06) *p* = 0.001	0.01 (−0.18 to 0.19) *p* = 0.954	−0.21 (−0.36 to −0.06) *p* = 0.005

Linear mixed model: gestational age, postnatal age, sex, state, imaging depth and birth mode were included as fixed effects. Imaging depth was categorized into four and three groups for arterial and venous signal respectively. Birth mode was categorized as spontaneous vaginal delivery, assisted vaginal delivery and cesarian section. Venous data are log-transformed. LFO, low frequency oscillations; PI, pulsatility index; PSV, peak systolic velocity; RI, resistive index; Vmean, mean velocity.

**Table 4:** Results from a linear mixed model to evaluate the association between cerebral hemodynamics and depth of the measurement.

Imaging depth	LFO index (%)	PSV (cm/s)	Vmean (cm/s)	RI	PI
Arterial	Estimate (95% CI)	Estimates (95% CI)	Estimate (95% CI)	Estimate (95% CI)	Estimate (95% CI)
15–20 (ref < 15) mm	0.04 (−0.30 to 0.37) *p* = 0.838	−0.24 (−0.81 to 0.34) *p* = 0.417	−0.14 (−0.50 to 0.22) *p* = 0.430	0.00 (−0.01 to 0.01) *p* = 0.696	0.00 (−0.02 to 0.02) *p* = 0.864
20–25 (ref < 15) mm	−0.19 (−0.52 to 0.14) *p* = 0.258	0.68 (0.09 to 1.28) *p* = 0.024	0.34 (−0.03 to 0.71) *p* = 0.069	0.02 (0.02 to 0.03) *p* < 0.001	0.07 (0.05 to 0.09) *p* < 0.001
>25 (ref < 15) mm	−0.60 (−0.92 to −0.28) *p* < 0.001	3.10 (2.52 to 3.67) *p* < 0.001	1.79 (1.43 to 2.15) *p* < 0.001	0.03 (0.02 to 0.04) *p* < 0.001	0.09 (0.07 to 0.10) *p* < 0.001
**Venous (log-transformed)**					
8–9 (ref < 8) mm	0.18 (−0.02 to 0.37) *p* = 0.074	0.37 (0.22 to 0.51) *p* < 0.001	0.34 (0.20 to 0.48) *p* < 0.001	0.00 (−0.09 to 0.08) *p* = 0.932	−0.01 (−0.11 to 0.09) *p* = 0.893
>9 (ref < 8) mm	0.18 (0.04 to 0.32) *p* = 0.011	0.19 (0.00 to 0.37) *p* = 0.046	0.15 (−0.04 to 0.35) *p* = 0.126	0.16 (0.04 to 0.28) *p* = 0.011	0.18 (0.04 to 0.32) *p* = 0.012

Linear mixed model: gestational age, postnatal age, sex, state, imaging depth and birth mode were included as fixed effects. Imaging depth was categorized into four and three groups for arterial and venous signal respectively. Birth mode was categorized as spontaneous vaginal delivery, assisted vaginal delivery and cesarian section. Venous data are log-transformed. LFO, low frequency oscillations; PI, pulsatility index; PSV, peak systolic velocity; RI, resistivity index; Vmean, mean velocity.

**Table 5:** Results from a linear mixed model to evaluate the association between cerebral hemodynamics and birth mode.

Birth mode (ref = spontaneous VD)	Assisted VD	C-section
Arterial signals	Estimate (95% CI)	Estimate (95% CI)
LFO index	−0.28 (−1.11 to 0.55) *p* = 0.503	0.06 (−0.46 to 0.57) *p* = 0.828
PSV	−1.18 (−2.32 to −0.05) *p* = 0.041	−0.44 (−1.06 to 0.19) *p* = 0.168
Vmean	−0.56 (−1.27 to 0.14) *p* = 0.116	−0.35 (−0.73 to 0.04) *p* = 0.077
RI	−0.03 (−0.05 to −0.02) *p* < 0.001	0.00 (−0.01 to 0.01) *p* = 0.539
PI	−0.09 (−0.15 to −0.04) *p* = 0.001	0.00 (−0.03 to 0.03) *p* = 0.871
**Venous signals (log-transformed)**		
LFO index	0.03 (−0.22 to 0.28) *p* = 0.789	0.11 (−0.04 to 0.26) *p* = 0.154
PSV	0.36 (0.02 to 0.71) *p* = 0.041	0.01 (−0.17 to 0.18) *p* = 0.923
Vmean	0.39 (0.03 to 0.75) *p* = 0.035	0.01 (−0.17 to 0.19) *p* = 0.893
RI	−0.39 (−0.59 to −0.19) *p* < 0.001	0.01 (−0.10 to 0.11) *p* = 0.920
PI	−0.43 (−0.65 to −0.20) *p* < 0.001	0.00 (−0.12 to 0.12) *p* = 0.979

* ref = spontaneous VD. Linear mixed model: gestational age, postnatal age, sex, state, imaging depth and birth mode were included as fixed effects. Imaging depth was categorized into four and three groups for arterial and venous signals respectively. Venous data are log-transformed. LFO, low frequency oscillations; PI, pulsatility index; PSV, peak systolic velocity; RI, resistive index; C-section, caesarian section; VD, vaginal delivery; Vmean, mean velocity.

**Figure 2:**
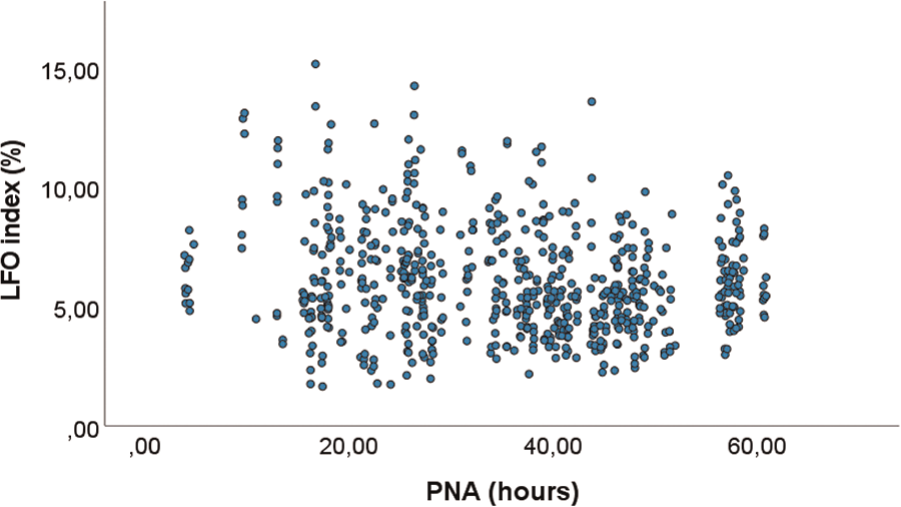
The relationship between arterial low frequency oscillations and postnatal age. The arterial low frequency oscillations (LFO) index (*y*-axis) is plotted against postnatal age (PNA) (*x*-axis).

In the univariable analyses (Table 2), mean arterial PSV and Vmean increased significantly from day one to day two. The following cerebral velocity measurements decreased significantly from day one to day two: mean arterial LFO index (Table 2 and Figure 2), and venous mean PSV, Vmean, RI and PI (Table 2). But for venous RI and PI the means at day two were only slightly smaller than on day one.

The results were similar after adjusting for factors that might influence CBF velocities; GA, PNA, sex, state, imaging depth and birth mode (Table 3). The mean arterial LFO index decreased with 0.92 percent points per postnatal day (95% CI: −1.31 to −0.53, *p* < 0.001) after adjusting for the other factors. The mean venous LFO index did not change significantly with increasing PNA (Table 3). The mean arterial RI and PI were significantly higher during sleep than wakefulness, while the opposite was true for mean venous RI and PI (Table 3).

**Figure 3:**
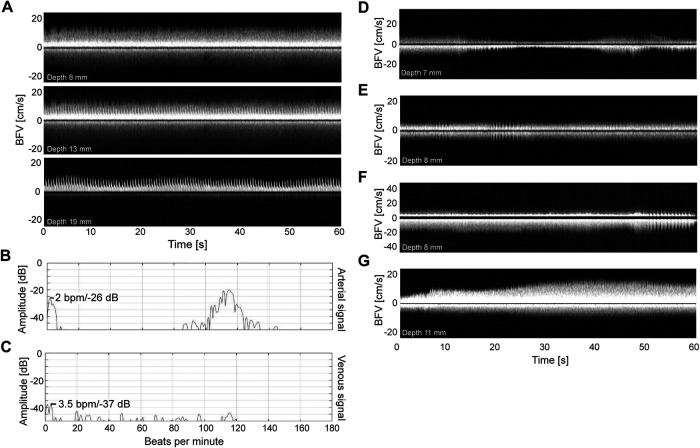
Examples of venous signals and the relationship between arterial and venous signals. (**A**) the Doppler velocity curves from a vein at 8 mm depth (upper panel), an artery at 19 mm depth (lower panel), and their relation to each other at 13 mm depth (middle panel). (**B,C**) the frequency spectrums of the vein (8 mm) and artery (19 mm) with beats/min along the *x*-axis and amplitude (decibel) along the *y*-axis. (**D–G**) Five typical examples of venous velocity measures. Each recording is from different neonatal subjects. Example 3**D,E** is recorded during sleep and 3**F,G** is recorded during wakefulness. The *x*-axis represents time of recording (seconds) and the *y*-axis represents velocity (cm/second).

Figures 3A–C demonstrates the relationship between blood flow velocity measured from a vein and a nearby artery. Figure 3A upper/middle/lower panel shows venous/both/arterial blood flow velocity respectively. As best seen in the middle panel, it appears that arterial pulsation leads to subsequent variations in venous blood flow. In the given example, there was more diversity in the venous frequency spectrum (3C) than the arterial frequency spectrum (3B). Figures 3D–G illustrate representative examples of blood flow velocity in proximal veins. High grade fluctuations are seen in examples 3E,F, but the speed never dropped to zero cm/second.

The intraclass correlation coefficient for inter-observer reliability was 0.98 for PI (95% CI: 0.97 to 0.99), 1.00 for the LFO index (95% CI: 0.99 to 1.00), 0.99 for Vmean (95% CI: 0.97 to 1.00), and 0.99 for PSV (95% CI: 0.97 to 0.99). The intraclass correlation coefficient for intra-observer reliability was 0.99 for PI (95% CI: 0.99 to 1.00), 0.99 for the LFO index (95% CI: 0.98 to 0.99), 1.00 for Vmean (95% CI: 0.99 to 1.00) and 1.00 for PSV (95% CI: 0.99 to 1.00).

### Safety and feedback from parents

The NeoDoppler probe did not cause any local adverse effects. The mechanical index ranged from 0.10 to 0.13 at the skin surface, dropping to 0.05 and 0.03 at two and three cm depth. With the intermittent recordings utilized—1 min on and 1 min off—the thermal index ranged from 0.14 to 0.39 degrees at the skin surface. Furthermore, due to the plane wave imaging, the maximum temperature increase dropped to 0.13 and 0.08 degrees at two and three cm depth respectively.

Parents of all except one neonate answered the questionnaire. The parents agreed that NeoDoppler did not seem to cause any discomfort (97.1% completely and 2.9% partly) or pain (100% completely agreed). Almost all the parents agreed that that NeoDoppler did not influence care (totally agree 91.4%, partly agree 5.7% and do not know 2.9%) or breastfeeding (totally agree 88.6%, partly agree 5.7% and do not know 5.7%).

## Discussion

In the present study, NeoDoppler was used to evaluate cerebral hemodynamics on the first and second day of life in healthy neonates born at term. We describe normal physiologic variations of arterial and venous CBF velocities. We also propose a new, feasible LFO index that is not influenced by the size of the vessel and the Doppler insonation angle and therefore could be practical in a monitoring setting. The LFO index is calculated as the standard deviation in percent of the mean blood velocity value over 1 min. This is the first work to describe venous LFO in neonates.

### Arterial LFO

This study indicates that arterial LFO decreases during the first 2 days of life; this decrease most likely represents normal physiological changes related to the transitional period. Previous studies of LFO in sick and healthy neonates using NIRS or US Doppler are generally small, and most of them are published more than 20 years ago (5–14). A longitudinal study including 30 preterm neonates found an inverse relationship between LFO and PNA (6). At the time, LFO in neonates was thought to represent an immature cerebral autoregulatory system, that was dampened with age (5, 6). In contrast, in a cross-sectional study including 15 preterm and term neonates, LFO was found to be independent of PNA (11). In the latter study, oscillations were measured over a longer time scale (10 min) than previous studies, which also captures very LFO (approximately 0.02 Hz) (11). In a longitudinal study including 10 preterm infants, the same research group found that although LFO was independent of PNA, LFO tended to be reduced in the early postnatal period (14). Similarly, Menke et al. (10) found that preterm neonates had few LFO directly after birth, and that the presence of LFO increased significantly during the first 96 h. This might indicate an initially impaired autoregulation in preterm neonates in the period they are known to be in greatest risk of intracranial complications (2, 10, 11). Physiological changes in CBF beyond the transitional period cannot be assumed based on our study.

In the present study, the arterial LFO index was similar during sleep and wakefulness. In contrast, Ferrari et al., using electroencephalography (EEG) to assess sleep states, found a larger amplitude of LFO in quiet sleep compared to active sleep in term born neonates (7). The LFO index in this study (Tables 1, 2) showed much lower values (range 2%–15%) than the percentage amplitudes reported by Ferrari et al. (range 12%–32%) (7), which can be explained by the differences in the measurement method.

### Arterial blood flow velocities and indices

In correspondence with other studies, the arterial CBF velocities increased from day one to day two of life (25). We were not able to detect the physiological drop in arterial RI and PI that occurs shortly after birth (25). This is probably due to the timing of the US recordings, as most of the neonates (77%) were older than 24 h at inclusion (25). As has previously been found, behavioral state influenced arterial PI, but not arterial Vmean and PSV (7).

The mean arterial CBF velocities, RI, and PI were lower than what has been found in other studies (25–28). The NeoDoppler system lacks a grayscale 2D-imaging to optimize the angle of insonation, which could have underestimated the velocity measurements. However, even with appropriate angle correction, increased beam-flow angles can cause a significant overestimation of flow velocity in other Doppler US systems (29). The present study includes measurements from arteries closer to the surface (53% imaging depth <20 mm). The findings are in line with the findings by Ecury-Goossen (30) who suggest that RI values for smaller arteries are likely to be lower than what has been reported for the ACA. In the present study, the CBF velocities, RI, and PI increased with longer distance from the US probe. It is also likely that the NeoDoppler US probe give less compression of the anterior fontanelle than traditionally handheld Doppler systems. Compression of the anterior fontanelle have been shown to give higher values of RI (27). The Doppler-data is averaged over several one-minute recordings (HR above 100 beats/min), while previous studies typically average over three cardiac cycles (26, 31, 32). With the beat-to-beat variation that is demonstrated in the present study, averaging over only three beats may be insufficient to measure the true mean value.

### Venous cerebral blood flow characteristics

We observed high-grade flow fluctuations in relatively proximal veins (range 7–11 mm). Large flow fluctuations with movement, crying and no apparent reason have previously been observed in the transverse and superior sagittal sinus (33, 34). The flow patterns in these deep cerebral veins are according to an old study normally less variable with a more flat pattern (34). In neonates with birth weight less than 1,000 g, high-grade fluctuations in the internal cerebral vein have been related to an increased risk of intraventricular hemorrhage (24). Compared to previous reported values from the transverse and superior sagittal sinus (33, 35, 36), the current paper report lower values for venous CBF velocities, RI and PI, which is probably due to the lack of angle correction and measurement in different veins.

### Study limitations and strengths

The main limitations in this study were a relatively low number of included neonates, the lack of recordings during the very first hours of life and the short follow-up time. As we only did measurements on two time periods during the transition phase, we were not able to cover the full dynamic process that happens during transition (37). However, compared to snapshot measurements using standard Doppler US, we have been able to perform numerous measurements over time for each patient to capture the physiologic cyclic variations over time. To be able to capture very LFO it would have been necessary to record CBF velocities in even longer time sequences than 1 min, and this will be explored in future studies. We did not obtain information regarding maternal factors, e.g., smoking, obesity etc., that could have influenced neonatal arterial and venous CBF.

Strengths were that this study included healthy neonates with demographics comparable to the national averages (38). The neonates could be together with their parents and/or breastfeed during examinations, and the NeoDoppler probe did not seem to cause discomfort. While recording with the NeoDoppler probe, it is possible to lift the neonates to comfort them, as demonstrated in a previous tilt test (39). The possibility for continuous monitoring of arterial and venous CBF velocities in several depths simultaneously is unique for NeoDoppler. Trends and relative changes in velocities and indices in several depths over time may provide a better insight into cerebrovascular hemodynamics. The excellent inter- and intra-observer reproducibility of velocity measures—an expected finding due to the automatic tracing—and need for only minor manual adjustments, suggests that NeoDoppler will give similar results when analyzed by different users. Furthermore, the broad beam that simultaneously covers several vessels makes it easier to use than conventional US. Moreover, fixation of the probe to receive Doppler signals does not require a trained US operator.

### Conclusion and future perspectives

The results indicate that arterial LFO decrease during the first 2 days of life in healthy neonates. This decrease most likely represents normal physiological changes related to the transitional period. A similar decrease for venous LFO was not found.

In a future clinical bedside monitoring setting, each beat-to-beat measure (such as PI, RI, HR, PSV) can be displayed with approximately 4 s delay, while the LFO index is calculated and displayed each minute. Future studies with longitudinal real-time recordings are necessary to evaluate whether NeoDoppler can be used as a tool to detect early signs of disease and/or deterioration of disease, e.g., in neonates admitted with suspected infection (40). There is also a need of future studies on sick and premature neonates are to validate NeoDoppler as a tool to improve outcomes in these patients. Doppler-derived parameters obtained during conventional “snapshot” US have so far not been able to predict neurological outcome in preterm infants (41). However, the possibility to measure Doppler parameters continuously and over time during a diagnostic treatment course, offers new possibilities including the assessment of LFO in the arterial as well as the venous system. Additional knowledge can be gained in future studies by using different monitoring methods such as NeoDoppler and NIRS simultaneously. Assessment of venous CBF may be a promising new approach in future studies on the relation between CBF and brain injury in preterm infants, as it is less influenced by vascular tone or shunts (41). Whether LFO alone or preferably in combination with invasive BP measurements could serve as an early marker of impaired autoregulation, remains to be studied.

## Data Availability

The datasets presented in this study can be found in online repositories. The names of the repository/repositories and accession number(s) can be found below: https://data.mendeley.com//datasets/b7ygpbk72t/1.

## References

[1] Rhee CJ da Costa CS Austin T Brady KM Czosnyka M Lee JK. Neonatal cerebrovascular autoregulation. Pediatr Res. (2018) 84(5):602–10. 10.1038/s41390-018-0141-6 30196311 PMC6422675

[2] Giesinger RE McNamara PJ. Hemodynamic instability in the critically ill neonate: an approach to cardiovascular support based on disease pathophysiology. Semin Perinatol. (2016) 40(3):174–88. 10.1053/j.semperi.2015.12.005 26778235

[3] Singh Y Villaescusa JU da Cruz EM Tibby SM Bottari G Saxena R Recommendations for hemodynamic monitoring for critically ill children-expert consensus statement issued by the cardiovascular dynamics section of the European society of paediatric and neonatal intensive care (espnic). Crit Care. (2020) 24(1):620. 10.1186/s13054-020-03326-2 33092621 PMC7579971

[4] Rickards CA Tzeng YC. Arterial pressure and cerebral blood flow variability: friend or foe? A review. Front Physiol. (2014) 5:120. 10.3389/fphys.2014.00120 24778619 PMC3985018

[5] Anthony MY Evans DH Levene MI. Neonatal cerebral blood flow velocity responses to changes in posture. Arch Dis Child. (1993) 69(3):304–8. 10.1136/adc.69.3_spec_no.304 8215571 PMC1029498

[6] Coughtrey H Rennie JM Evans DH. Postnatal evolution of slow variability in cerebral blood flow velocity. Arch Dis Child. (1992) 67(4):412–5. 10.1136/adc.67.4_spec_no.412 1586181 PMC1590476

[7] Ferrarri F Kelsall AW Rennie JM Evans DH. The relationship between cerebral blood flow velocity fluctuations and sleep state in normal newborns. Pediatr Res. (1994) 35(1):50–4. 10.1203/00006450-199401000-00012 8134199

[8] Kato I Kusaka T Nishida T Koyano K Nakamura S Nakamura M Extrauterine environment influences spontaneous low-frequency oscillations in the preterm brain. Brain Dev. (2013) 35(1):17–25. 10.1016/j.braindev.2012.03.007 22534236

[9] Livera LN Wickramasinghe YA Spencer SA Rolfe P Thorniley MS. Cyclical fluctuations in cerebral blood volume. Arch Dis Child. (1992) 67(1):62–3. 10.1136/adc.67.1_spec_no.62 1536589 PMC1590332

[10] Menke J Michel E Hillebrand S von Twickel J Jorch G. Cross-spectral analysis of cerebral autoregulation dynamics in high risk preterm infants during the perinatal period. Pediatr Res. (1997) 42(5):690–9. 10.1203/00006450-199711000-00023 9357945

[11] Michel E Zernikow B Steck J Kohlmann G von Siebenthal K Hirano S Cyclic variation pattern of cerebral blood flow velocity and postconceptional age. Eur J Pediatr. (1994) 153(10):751–5. 10.1007/BF01954493 7813534

[12] Taga G Konishi Y Maki A Tachibana T Fujiwara M Koizumi H. Spontaneous oscillation of oxy- and deoxy- hemoglobin changes with a phase difference throughout the occipital cortex of newborn infants observed using non-invasive optical topography. Neurosci Lett. (2000) 282(1-2):101–4. 10.1016/s0304-3940(00)00874-0 10713406

[13] von Siebenthal K Beran J Wolf M Keel M Dietz V Kundu S Cyclical fluctuations in blood pressure, heart rate and cerebral blood volume in preterm infants. Brain Dev. (1999) 21(8):529–34. 10.1016/s0387-7604(99)00062-5 10598053

[14] Zernikow B Michel E Kohlmann G Steck J Schmitt RM Jorch G. Cerebral autoregulation of preterm neonates–a non-linear control system? Arch Dis Child Fetal Neonatal Ed. (1994) 70(3):F166–73. 10.1136/fn.70.3.f166 8198408 PMC1061034

[15] Brady KM Lee JK Kibler KK Easley RB Koehler RC Shaffner DH. Continuous measurement of autoregulation by spontaneous fluctuations in cerebral perfusion pressure: comparison of 3 methods. Stroke. (2008) 39(9):2531–7. 10.1161/STROKEAHA.108.514877 18669896 PMC2566962

[16] Brady KM Lee JK Kibler KK Smielewski P Czosnyka M Easley RB Continuous time-domain analysis of cerebrovascular autoregulation using near-infrared spectroscopy. Stroke. (2007) 38(10):2818–25. 10.1161/STROKEAHA.107.485706 17761921 PMC2377358

[17] Vermeij A Meel-van den Abeelen AS Kessels RP van Beek AH Claassen JA. Very-low-frequency oscillations of cerebral hemodynamics and blood pressure are affected by aging and cognitive load. Neuroimage. (2014) 85(Pt 1):608–15. 10.1016/j.neuroimage.2013.04.107 23660026

[18] Schroeter ML Bucheler MM Preul C Scheid R Schmiedel O Guthke T Spontaneous slow hemodynamic oscillations are impaired in cerebral microangiopathy. J Cereb Blood Flow Metab. (2005) 25(12):1675–84. 10.1038/sj.jcbfm.9600159 15931161

[19] Aaslid R Newell DW Stooss R Sorteberg W Lindegaard KF. Assessment of cerebral autoregulation dynamics from simultaneous arterial and venous transcranial Doppler recordings in humans. Stroke. (1991) 22(9):1148–54. 10.1161/01.str.22.9.1148 1926257

[20] Strik C Klose U Kiefer C Grodd W. Slow rhythmic oscillations in intracranial csf and blood flow: registered by mri. Acta Neurochir Suppl. (2002) 81:139–42. 10.1007/978-3-7091-6738-0_36 12168286

[21] Howlett JA Northington FJ Gilmore MM Tekes A Huisman TA Parkinson C Cerebrovascular autoregulation and neurologic injury in neonatal hypoxic-ischemic encephalopathy. Pediatr Res. (2013) 74(5):525–35. 10.1038/pr.2013.132 23942555 PMC3954983

[22] Lee JK Kibler KK Benni PB Easley RB Czosnyka M Smielewski P Cerebrovascular reactivity measured by near-infrared spectroscopy. Stroke. (2009) 40(5):1820–6. 10.1161/STROKEAHA.108.536094 19286593

[23] Vik SD Torp H Follestad T Stoen R Nyrnes SA. Neodoppler: new ultrasound technology for continous cerebral circulation monitoring in neonates. Pediatr Res. (2020) 87(1):95–103. 10.1038/s41390-019-0535-0 31404920 PMC6960092

[24] Ikeda T Amizuka T Ito Y Mikami R Matsuo K Kawamura N Changes in the perfusion waveform of the internal cerebral vein and intraventricular hemorrhage in the acute management of extremely low-birth-weight infants. Eur J Pediatr. (2015) 174(3):331–8. 10.1007/s00431-014-2396-1 25169064

[25] Stritzke A Murthy P Kaur S Kuret V Liang Z Howell S Arterial flow patterns in healthy transitioning near-term neonates. BMJ Paediatr Open. (2019) 3(1):e000333. 10.1136/bmjpo-2018-000333 30957024 PMC6422249

[26] Aranha CA Lederman HM Segre CA. Color Doppler evaluation of the influence of type of delivery, sex, postnatal age and time post feeding on full term healthy newborns cerebral blood flow. Arq Neuropsiquiatr. (2009) 67(2B):463–73. 10.1590/s0004-282×2009000300017 19623445 10.1590/s0004-282x2009000300017

[27] Elmfors AF Sandgren T Ford K Rosenberg J Ringertz H Barth RA Normal values of the resistivity Index of the pericallosal artery with and without compression of the anterior fontanelle. Pediatr Radiol. (2019) 49(5):646–51. 10.1007/s00247-019-04347-y 30712160

[28] Forster DE Koumoundouros E Saxton V Fedai G Holberton J. Cerebral blood flow velocities and cerebrovascular resistance in normal-term neonates in the first 72 hours. J Paediatr Child Health. (2018) 54(1):61–8. 10.1111/jpc.13663 28845537

[29] Park MY Jung SE Byun JY Kim JH Joo GE. Effect of beam-flow angle on velocity measurements in modern Doppler ultrasound systems. Am J Roentgenol. (2012) 198(5):1139–43. 10.2214/AJR.11.7475 22528905

[30] Ecury-Goossen GM Raets MM Camfferman FA Vos RH van Rosmalen J Reiss IK Resistive indices of cerebral arteries in very preterm infants: values throughout stay in the neonatal intensive care unit and impact of patent ductus arteriosus. Pediatr Radiol. (2016) 46(9):1291–300. 10.1007/s00247-016-3615-x 27259991 PMC4943974

[31] Fenton AC Shortland DB Papathoma E Evans DH Levene MI. Normal range for blood flow velocity in cerebral arteries of newly born term infants. Early Hum Dev. (1990) 22(2):73–9. 10.1016/0378-3782(90)90081-s 2364906

[32] Yoshida H Yasuhara A Kobayashi Y. Transcranial Doppler sonographic studies of cerebral blood flow velocity in neonates. Pediatr Neurol. (1991) 7(2):105–10. 10.1016/0887-8994(91)90005-6 2059249

[33] Baytur YB Tarhan S Uyar Y Ozcakir HT Lacin S Coban B Assessment of fetal cerebral arterial and venous blood flow before and after vaginal delivery or cesarean section. Ultrasound Obstet Gynecol. (2004) 24(5):522–8. 10.1002/uog.1749 15459931

[34] Cowan F Thoresen M. Changes in superior sagittal sinus blood velocities due to postural alterations and pressure on the head of the newborn infant. Pediatrics. (1985) 75(6):1038–47. 10.1542/peds.75.6.1038 3889817

[35] d'Orey C Mateus M Guimaraes H Ramos I Melo MJ Silva J Neonatal cerebral Doppler: arterial and venous flow velocity measurements using color and pulsed Doppler system. J Perinat Med. (1999) 27(5):352–61. 10.1515/JPM.1999.048 10642955

[36] Liu LY Hong JL Wu CJ. A preliminary study of neonatal cranial venous system by color Doppler. Biomed Res Int. (2019) 2019:7569479. 10.1155/2019/7569479 31183374 PMC6512013

[37] Alderliesten T Dix L Baerts W Caicedo A van Huffel S Naulaers G Reference values of regional cerebral oxygen saturation during the first 3 days of life in preterm neonates. Pediatr Res. (2016) 79(1-1):55–64. 10.1038/pr.2015.186 26389823

[38] http://statistikkbank.fhi.no/mfr/.

[39] Jarmund AH Odegard SS Torp H Nyrnes SA. Effects of tilt on cerebral hemodynamics measured by neodoppler in healthy neonates. Pediatr Res. (2021) 90:888–95. 10.1038/s41390-020-01354-w 33504967 PMC8566239

[40] Ratnaparkhi CR Bayaskar MV Dhok AP Bhende V. Utility of Doppler ultrasound in early-onset neonatal sepsis. Indian J Radiol Imaging. (2020) 30(1):52–8. 10.4103/ijri.IJRI_265_19 32476750 PMC7240890

[41] Camfferman FA de Goederen R Govaert P Dudink J van Bel F Pellicer A Diagnostic and predictive value of Doppler ultrasound for evaluation of the brain circulation in preterm infants: a systematic review. Pediatr Res. (2020) 87(Suppl 1):50–8. 10.1038/s41390-020-0777-x 32218536 PMC7098887

